# Rhinocerebral mucormycosis: a rare infection

**DOI:** 10.11604/pamj.2017.26.143.12047

**Published:** 2017-03-14

**Authors:** Moncef Sellami, Malek Mnejja

**Affiliations:** 1 Department of Otorhinolaryngology-Head and Neck Surgery Habib Bourguiba University Hospital, Sfax, Tunisia; 2 Sfax Medical School, University of Sfax, Sfax, Tunisia

**Keywords:** Diabetes mellitus, rhinocerebral mucormycosis, fungal infection

## Image in medicine

Rhinocerebral mucormycosis, also known as zygomycosis, is an acute opportunistic fungal infection with high associated mortality rates. There are some predisposing factors for mucormycosis such as hematological malignancies, severe burns, neutropenia, diabetes mellitus, and the use of corticosteroids. Treatment of rhinocerebral mucormycosis consists of treating the predisposing disease, aggressive surgical debridement and systemic antifungal therapy. We present the case of a 55-year-old diabetic patient treated with insulin who was admitted to the emergency department for a headache and left purulent rhinorrhea for 3 days. The patient was hospitalized for diabetic ketoacidosis, and intravenous insulin infusion therapy and intravenous antibiotherapy were administered. The day after hospitalization the patient developed a ptosis of the left eye associated with edema of the upper eyelid and the left cheek. The nasal endoscopy showed necrotic tissue in the left middle meatus and the middle turbinate associated with profuse purulent discharge. The culture of the nasal swab identified a *Rhizopus oryzae* and the diagnosis of rhino-cerebral mucormycosis was retained. he patient was put on amphotericin B (1 mg/kg/daily) under medical care in the hospital. The patient underwent a functional endoscopic sinus surgery with debridement of sinuses, ethmoidectomy and left middle meatal antrostomy. Histopathological findings from the biopsy of the maxillary sinus presented aseptate hyphae in necrotic tissue. The outcome was fatal on the third day of hospitalization, despite appropriate reanimation measures.

**Figure 1:**
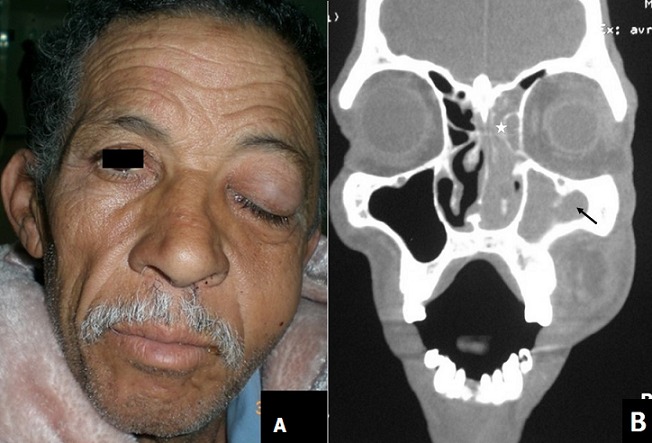
(A) clinical examination at the second day of hospitalization found a ptosis of the left eye associated with edema of the left cheek; (B) coronal computed tomography scan showed soft tissue opacification of the left maxillary (arrow) and ethmoid sinuses (star) without orbital involvement

